# UMPlex™: a targeted next-generation sequencing primer design workflow

**DOI:** 10.1186/s12985-025-02831-6

**Published:** 2025-07-05

**Authors:** Dachuan Lin, Xiaomin Zhang, Dan Wang, Tongyingzi Liu, Tong Li, Yingluan Zhang, Lihua Li, Yi Huang, Yongchao Guo, Renli Zhang, Xinchun Chen, Tiejian Feng

**Affiliations:** 1 Hainan Medical University-The University of Hong Kong Joint Laboratory of Tropical Infectious Diseases, Hainan Medical University & Key Laboratory of Tropical Translational Medicine of Ministry of Education, Hainan Medical University, Haikou, China; 2 Guangdong Provincial Key Laboratory of Regional Immunity and Diseases, Department of Pathogen Biology, Shenzhen University Medical School, Shenzhen, 518060 China; 3 Shenzhen Center for Disease Control and Prevention, 8 Longyuan Rd, Shenzhen, 518055 China; 4 Shenzhen Uni-Medica Technology Co., Ltd., Block 6th, Liuxian Culture Park, Shenzhen, China

**Keywords:** Targeted next-generation sequencing, Personalized primer design, Clinical surveillance, Influenza-like patients

## Abstract

We have developed a tailored next-generation sequencing (tNGS) panel, employing our innovative UMPlex™ primer design workflow, to enhance pathogen identification in clinical diagnostics. Through iterative experimentation and rigorous validation, we refined the primer design by excluding those with insufficient specificity or efficiency. To mitigate amplification challenges arising from pathogenic mutations, we implemented a strategy of using a minimum of two primer pairs per pathogen, ensuring redundancy and robust detection. Validation using clinical samples showcased high specificity and efficacy, with 11 cultured pathogens isolated exclusively. In a study involving 107 positive respiratory samples, tNGS outperformed the TaqMan Array, detecting a higher number of pathogens in patients with influenza-like symptoms of unknown etiology. Additionally, tNGS yielded higher read counts for potentially pathogenic microorganisms and produced results consistent with metagenomic NGS, despite generating a reduced data volume. This approach not only improves detection rates but also offers a flexible tool for both clinical diagnostics and surveillance, particularly in the context of influenza-like illnesses.

**Supplementary Information:**

The online version contains supplementary material available at 10.1186/s12985-025-02831-6.

## Highlights

Rapid and accurate pathogen identification is essential for effective diagnosis and treatment of infectious diseases.

Targeted next-generation sequencing (tNGS) offers a revolutionary approach for comprehensive pathogen detection.

Our pioneering work introduces a systematic primer validation methodology, addressing amplification inconsistencies in tNGS designs.

This method has been successfully validated in influenza-like patients, demonstrating its efficacy and reliability.

**Supplementary Information:**

The online version contains supplementary material available at 10.1186/s12985-025-02831-6.

## Introduction

Annually, the global population faces a disconcerting mortality ranging from 291,243 to 645,832 respiratory-related deaths attributable to seasonal influenza, resulting in a mortality rate of 4.0 to 8.8 per 100,000 individuals [1]. It is universally acknowledged that the key point in averting infectious disease pandemics lies in early detection. Swift identification of outbreaks empowers public health systems to deploy proactive measures, such as quarantining and managing afflicted individuals at designated facilities or outbreak sites, thereby impeding the spread of infection [2]. In light of this critical need, numerous surveillance systems for emergent infectious diseases, including influenza, have been globally instituted [3]. However, extant systems narrowly monitor specific influenza viruses, overlooking a plethora of pathogens capable of inducing influenza-like illnesses. Influenza-like illnesses manifest a spectrum of clinical symptoms, from mild respiratory issues to severe lung damage, attributable to a diverse array of pathogens, including the notorious severe acute respiratory syndrome coronavirus 2 (SARS-CoV-2) [4, 5]. The concurrent occurrence of bacterial and viral infections amidst individuals exhibiting influenza-like symptoms is a commonplace clinical occurrence [6, 7]. Notably, the clinical presentations of influenza and streptococcal throat infections frequently overlap, prompting the prescription of antibiotics that may alleviate symptoms [8]. Given the multiplicity of respiratory pathogens underlying influenza-like illnesses, there is an urgent imperative for efficient, sensitive, and specific diagnostic tools capable of simultaneously detecting multiple pathogens, pivotal in epidemic control and the expeditious identification of causal agents in patients with influenza-like symptoms. In recent years, molecular-based pathogen detection systems have garnered research attention due to their expeditious nature. However, conventional molecular techniques such as qPCR assays present limitations, necessitating repeated testing for the detection of co-infections in respiratory diseases. Although multiplex commercial assays (e.g., TaqMan Array) can detect 10 + pathogens simultaneously, they lack comprehensive coverage for rare pathogens and antibiotic resistance genes, necessitating supplemental single-pathogen tests for suspected co-infections. For instance, while reverse-transcription quantitative PCR (RT-qPCR) has been established as the gold standard for diagnosing COVID-19 [9, 10], its pathogen coverage remains suboptimal. Specifically, RT-qPCR primarily targets SARS-CoV-2, offering limited coverage for other respiratory pathogens (e.g., bacteria, fungi) that may cause similar symptoms. The operational costs and time investments associated with sequential testing for primary and secondary suspected pathogens in surveillance scenarios further exacerbate the issue. Despite the widespread prevalence of influenza and its substantial impact on global health, the etiology of many patients with influenza-like illnesses remains inadequately elucidated [11]. Indeed, the etiological detection of respiratory symptoms has long been a formidable challenge, as identifying the underlying cause of infection proves arduous; even the most comprehensive prospective studies employing available microbiologic techniques generally fail to identify an etiological agent in more than 50% of cases [12, 13].

To address the issue of insufficient coverage in the pathogen spectrum, contemporary scholarly endeavors have predominantly focused on assessing the effectiveness of metagenomic Next-Generation Sequencing (mNGS) [14]. However, clinical samples inevitably contain a substantial amount of human nucleic acids, and the prevalence of humanized genes has constrained its applicability in pathogen diagnostic procedures, impacting both detection time and cost. Consequently, there has been a persistent pursuit for more efficient diagnostic tools capable of delivering rapid results across a wide array of pathogens while maintaining sensitivity and specificity. Recently, a transformative technology known as targeted Next-Generation Sequencing (tNGS) has emerged, offering the promise of swift and highly sensitive detection of pathogenic nucleic acids [15, 16]. While tNGS boasts significant cost and time advantages over mNGS, it requires a higher level of sophistication in primer design compared to techniques such as qPCR. This is the primary reason for its limited widespread adoption, primarily due to the complexities involved in primer design. As a result, numerous clinical practitioners favor the use of commercial products over crafting solutions tailored to their specific needs and contexts. This has led to a scarcity of tNGS reagent kits; for instance, there are currently no tNGS reagent kits specifically designed for patients exhibiting influenza-like symptoms. One of the objectives of this study is to demonstrate the potential of tNGS through a performance comparison between tNGS and mNGS, utilizing our designed tNGS approach. The development of diagnostic methodologies has encountered challenges related to primer inclusivity, specificity, and the amplification of multiple primer sets. This study delineates the design of primers for Influenza A and *Staphylococcus aureus* (*S. aureus*), show our tNGS primer design scheme -UMPlex™, and validates their efficacy in enabling the rapid and concurrent single-plex detection of various viral and bacterial respiratory pathogens. Furthermore, we propose a method to address amplification efficiency imbalances caused by genetic mutations. Based on UMPlex™, a reagent kit for tNGS covering 125 respiratory pathogens has been successfully formulated. This research culminates in the validation of our tNGS kit against alternative detection methods, underscoring its relevance for influenza surveillance and its contribution to advancing contemporary diagnostic solutions.

## Methods

### Primer design

We selected 330 gene fragments from 125 respiratory pathogens prevalent in China [17], including viruses, bacteria, fungi, and antibiotic resistance genes, based on their ubiquity in relevant literature and existing assays (Supplemental Table 1). After downloading reference genomes from NCBI, we identified conserved genomic regions to design a comprehensive primer library, aiming to meet amplification length criteria for standard strains like influenza A’s NP and M proteins [18]. Primer 3 software facilitated the design process, generating a primer pool for subsequent in silico evaluation [19].

### In silico analysis

To represent global etiology diversities, we validated our primer pool using the NCBI genome repository (May 2023 release), allowing a maximum of two mismatches and excluding mismatches within the 3’ terminal quintuple bases. We performed specificity checks via BLAST against the NCBI nr/nt database (November 17, 2022). Taxonomic categorization involved BLASTn analysis against the NCBI taxonomy database. Primer efficiency predictions were based on a detailed examination of “complete status” sequencing data from the Pathosystems Resource Integration Center (PATRIC). We set a coverage threshold of at least 95%, with all primers matching their targeted pathogen sequences at a 100% coverage rate. Primers were then ranked based on in silico inclusion, specificity, and efficiency, and those with the highest scores underwent further empirical validation. The process involved iterative primer refinement through replacement and reevaluation until satisfactory performance was achieved.

### Testing amplification uniformity

For quantitative evaluation of amplification homogeneity, we constructed plasmids representing each primer target, mixed them evenly, and subjected them to tNGS analysis. After 12 cycles of amplification, the number of reads per primer target served as an indicator of amplification uniformity.

### Strains and clinical specimens

To evaluate the specificity, sensitivity, and amplification efficiency of our primer set, we used collected samples, including viral and bacterial isolates, as well as clinical oropharyngeal swab specimens (with corresponding serum C-reactive protein (CRP) results). All specimens were obtained from the Shenzhen Centers for Disease Control and Prevention (CDC) in compliance with regulations protecting human subjects in research. Subtypes of seasonal influenza A virus strains were determined by the Shenzhen CDC. Influenza-like illness was defined based on specific symptoms and fever exceeding 37.5 °C. PCR was performed on oropharyngeal swabs to detect influenza and other respiratory viruses, and standard individual information was collected through a designated form [20].

### Total Nucleic Acids (TNA) extraction

TNA extraction followed the instructions provided by Shenzhen United Medical Technology for their nucleic acid extractor and accompanying reagents. Viral RNA was extracted using the RNeasy kit (Qiagen) according to the manufacturer’s protocol. Extracted viral nucleic acids were stored at −80 °C for future use.

### Validation of tNGS using clinical specimens

To assess the clinical applicability of tNGS, we compared its performance to RT-PCR assays conducted by the WHO and US CDC. Our evaluation included 107 oropharyngeal swab specimens previously tested positive for viruses causing influenza-like symptoms, such as influenza A, and 50 control group samples from individuals without such illnesses. The specimens comprised various types, including those tested negative for seasonal influenza A virus, influenza B, or SARS-CoV-2, as well as bronchoalveolar lavage fluid samples.

### Analytical sensitivity

The accuracy of sequence data from positive clones was confirmed using Sanger sequencing technology. A 10-fold dilution series of plasmid DNA, ranging from 500 copies/ml to 8 copies/ml, was prepared. Sample concentrations in copy number were determined using the Nanodrop 2000 to measure absorbance and original plasmid DNA concentrations. Two sets of experiments assessed analytical sensitivity: comparative analytical-sensitivity experiments and comparative limit-of-detection (LOD) experiments. The lowest detectable concentration for each assay was established as the highest dilution where all four replicates tested positive.

## Results

### Primer design and validation process

**Fig. 1:**
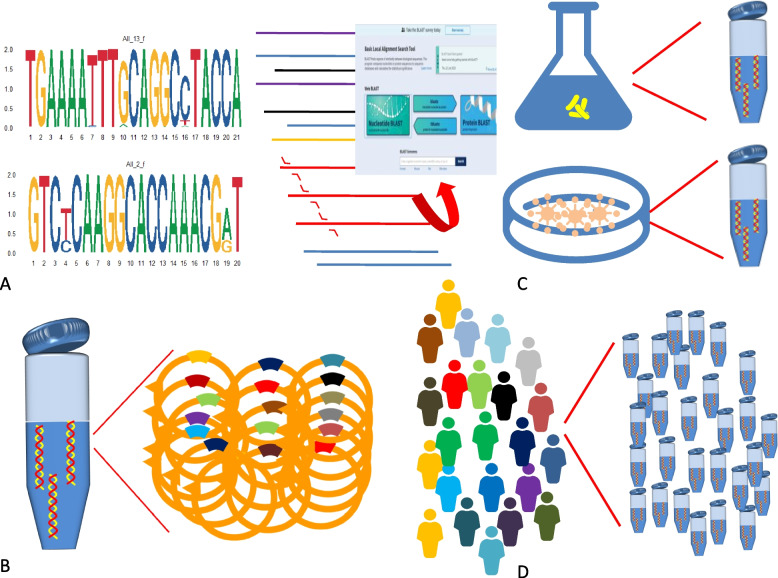
Schematic workflow of the design and validation pipeline for tNGS primers (UMPlex™)

We developed primer sets based on shared consensus sequences among various strains. Following their creation, these primers underwent a thorough evaluation process to ensure both specificity and inclusivity. Detailed bioinformatics analyses were employed to filter out any primers that did not meet our established criteria (Fig. 1A). Subsequently, we synthesized plasmid constructs to represent the genomic regions targeted by each primer and optimized primer concentrations to achieve consistent amplification efficiency (Fig. 1B). To assess the performance of the amplification system, specificity tests were conducted using nucleic acid data from pure microbial cultures (Fig. 1C) as well as clinical samples (Fig. 1D).

### Bioinformatic assessment and outcomes for screened primers

Given the extensive number of primers available, we concentrated on presenting the final, validated sets. These primers were subjected to comprehensive in silico evaluations to determine their coverage of specific genetic sequences. For each pathogen, multiple targets were selected to guarantee complete coverage, achieving a 100% coverage rate (Table 1; Supplemental Table 2). For example, primers designed for detecting influenza A virus and *S. aureus* achieved 100% coverage across datasets comprised of 130 influenza A virus and 1,342 *S. aureus* genomes sourced from the NCBI database.

**Table 1:** Evaluation of primer coverage via electronic PCR. This table presents an assessment of primer coverage using electronic PCR, exemplified by the cases of Influenza A virus and *S. aureus*. The detailed comparison results are presented in Supplemental Table 2

Pathogen	Primer_id	Inclus_match	Number_in_database	Coverage
*Influenza* A	PC-13	127	130	97.7%
	PC-2	128	130	98.5%
	Combined_coverage^a^	130	130	100%
*Staphylococcus aureus*	PC-16	1342	1342	100%
	PC-17	1342	1342	100%
	Combined_coverage^a^	1342	1342	100%

^a^For each pathogen, a “positive” record signifies the successful detection of at least one pair of primers

To ensure high specificity, we established a stringent threshold of 99.5%. Primers P13 and P2, aimed at influenza A virus detection, demonstrated exemplary specificity, aligning closely with their target sequences (Table 2; Supplemental Table 3).

**Table 2:** Evaluation of primer specificity via electronic PCR. This table illustrates the evaluation of primer specificity by comparing the statistical results following sequence identification with primer alignment, using data sourced from the NCBI NT (Nucleotide) database. The detailed comparison results are presented in Supplemental Table 3

Pathogen	Primer_id	True positive number	False positive number
Influenza A	PC-13	32,604	1^a^
	PC-2	49,975	0
*Staphylococcus aureus*	PC-16	1510	0
	PC-17	1507	0

^a^Indicates samples marked as ‘polluted’ in the NCBI NT database

### Uniformity of primer amplification

**Fig. 2:**
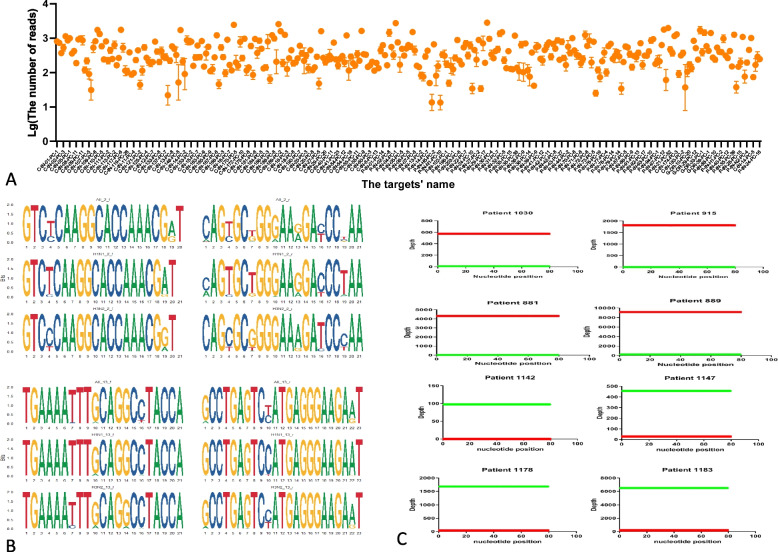
Evaluation of primer amplification uniformity. **A** This section presents the number of amplified reads per primer pair across three replicated experiments. The vertical axis denotes the logarithm (base 10) of the number of reads amplified, while the horizontal axis lists the names of the amplicons. The data points represent the mean number of amplified reads. **B** This section showcases a statistical motif involving two pairs of universal primers designed for electronic PCR targeting the Influenza A virus and its characteristic subtypes. The graphics were created utilizing the R package ggseqlogo [21]. **C** An illustrative example is provided, demonstrating the actual number of reads amplified by primers for typical subtypes of the Influenza A virus. The reads amplified by the PC-13 primer (targeting the M protein) are highlighted in red, whereas those amplified by the PC-2 primer (targeting the NP protein) are marked in green. The depth indicates the number of reads at each position. Patients 1030, 915, 881, and 889 are confirmed to carry the H1N1 virus, while patients 1142, 1147, 1178, and 1183 are identified as carriers of the H3N2 virus. Detailed statistical results are documented in Supplemental Tables 4–6

To assess the uniformity of amplification by the system, we mixed equal amounts of plasmids and selected primers whose read ratios clustered within a 0.2- to 5-fold range of the average (Fig. 2A). This validated that the tNGS system exhibits excellent amplification efficiency when the target sequences perfectly match the designed primers. However, actual pathogen sequences often contain mutations. For example, analysis of 38,433 influenza A virus genome sequences from various subtypes in the PATRIC database revealed variable mutation frequencies at positions targeted by the P13 primer (Fig. 2B). These mutations could theoretically reduce amplification efficiency. Nevertheless, our strategy of designing multiple primer pairs for different gene fragments of each pathogen ensured robust amplification: at least one primer pair consistently yielded effective amplification, even when other pairs were affected by mutations (Fig. 2C).

### Analytical sensitivity and repeatability of nucleic acid detection methods

We rigorously evaluated the lower limits of detection for quantitative PCR (qPCR) and tNGS by employing serial dilutions of nucleic acids obtained from pure cultured pathogens. A sample was considered positive only if it generated a complete amplification curve. The sensitivity analysis demonstrated that tNGS had a superior detection aptitude, identifying *S. aureus* at concentrations as low as 4 colony-forming units per milliliter (CFU/ml) (Table 3).

**Table 3:** Analytical sensitivity ranges and precision. Positive results were determined based on the recorded CT values in qPCR, with the corresponding actual CT values available in Supplemental Table 7 (Sheet 3)

	No. of detected samples for different organisms							
No. of detected samples CFU or PFU/ml	*S.aureus*				Influenza A			
	Qpcr	tNGS			Qpcr	tNGS		
		Pc-16	Pc-17	combine		Pc-13	Pc-2	combine
512	4/4	4/4	4/4	4/4	4/4	4/4	1/4	4/4
256	4/4	4/4	4/4	4/4	**4/4**	4/4	1/4	4/4
128	**4/4**	4/4	4/4	4/4	3/4	4/4	0/4	**4/4**
64	1/4	4/4	4/4	4/4	2/4	3/4	1/4	3/4
32	1/4	4/4	3/4	4/4	0/4	4/4	1/4	4/4
16	0/4	4/4	3/4	4/4	0/4	1/4	0/4	1/4
8	0/4	2/4	3/4	4/4	0/4	2/4	0/4	2/4
4	0/4	2/4	4/4	**4/4**	0/4	2/4	0/4	2/4

**Fig. 3:**
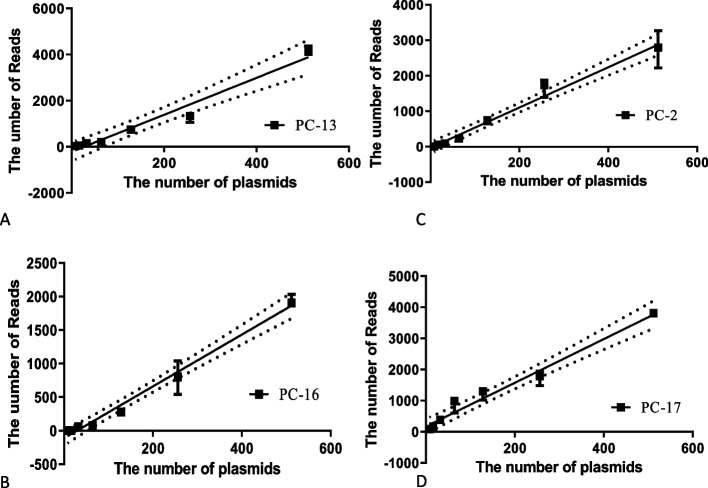
Assessment of primer amplification efficiency. **A** and **B** These sections illustrate the correlation between the number of plasmids per sample and the corresponding number of reads for primers targeting Influenza A virus. **C** and **D** These sections depict the relationship between the quantity of plasmids and the number of reads for primers targeting *S. aureus*. Detailed comparative results can be found in the corresponding actual read values in Supplemental Table 7 (Sheet 1)

The quantification accuracy, derived from nucleic acid dilutions of pure cultures, revealed a robust linear relationship between the number of amplifications and sample concentrations exceeding 100 copies/ml, further supported by rigorous statistical analyses (Fig. 3; Extended Fig. 1). Additionally, we confirmed consistent repeatability through thorough experimentation, with detailed results available in the Supplemental data (Supplemental Tables 8 and 9).

### Specificity of tNGS

The specificity of the tNGS system was rigorously evaluated by amplifying 11 pure cultured pathogens and analyzing 107 clinical samples. Among the pure cultured pathogens, no cross-reactivity with other influenza virus subtypes or respiratory viruses was observed (Table 4; Supplemental Table 10). For all 107 clinical samples with identified pathogens, tNGS produced results that aligned with known pathogen identities. Importantly, asymptomatic control samples exhibited significantly fewer reads compared to those from symptomatic individuals (Extended Fig. 2).

**Table 4:** Validation of amplification specificity using clinical samples from patients with confirmed pathogens

Pathogen^a^	Influenza A virus	Influenza B virus	SARS-CoV-2	Human coronavirus 229E	Human coronavirus OC43	Human respiratory syncytial virus	Human orthorubulavirus	Bocaparvovirus
Influenza A virus	**75**							
Influenza B virus		**3**						
SARS-CoV-2			**24**					
Human coronavirus 229E				**1**				
Human coronavirus OC43					**1**			
Human respiratory syncytial virus						**2**		
Human orthorubulavirus							**1**	
Bocaparvovirus								**1**

^a^The pathogen corresponding to the most reads detected by tNGS The corresponding actual reads are available in Supplemental Table 11

### Microorganism identification in influenza-like illness patients

**Fig. 4:**
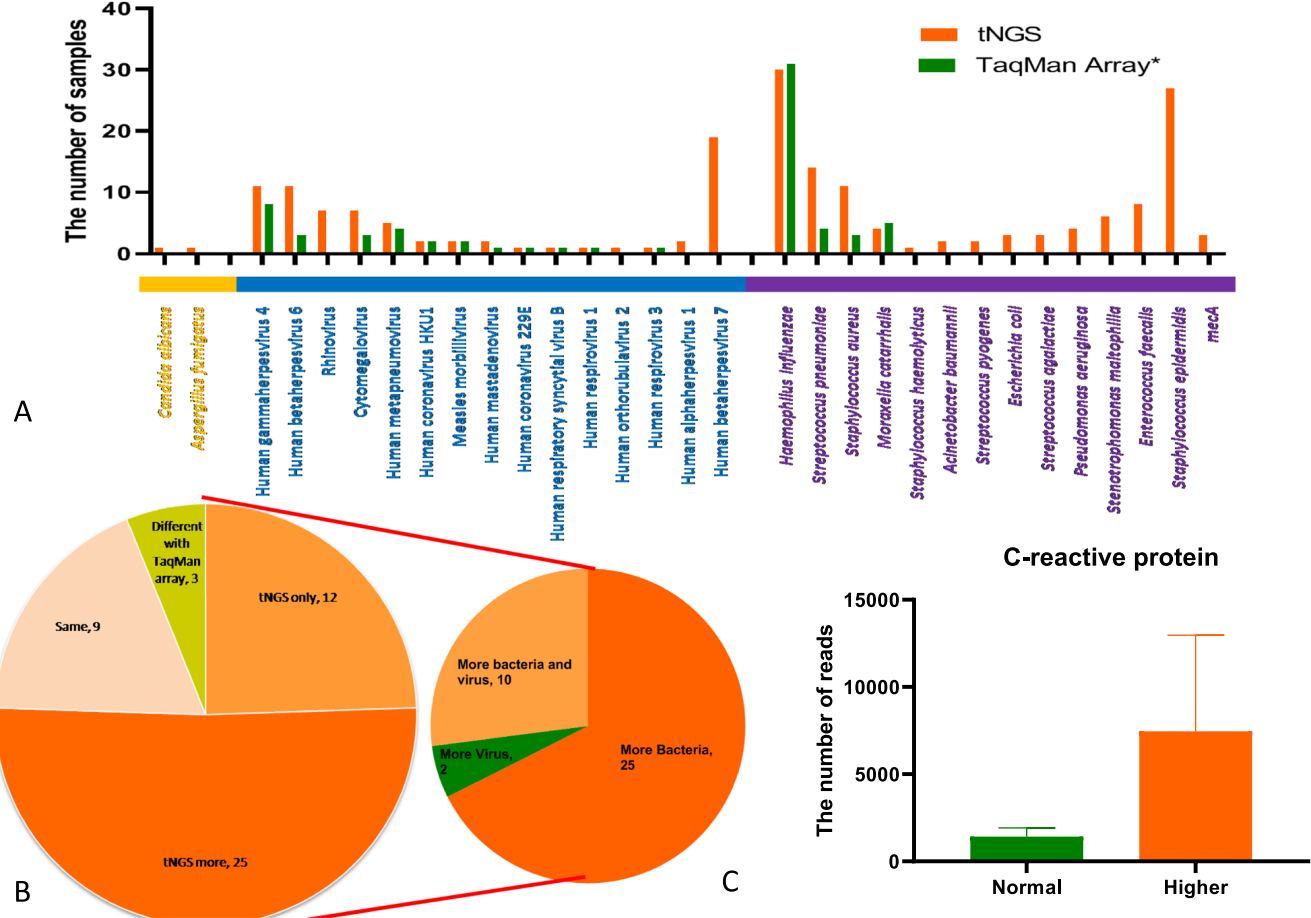
Comparison of tNGS and TaqMan array in influenza-like patients with non-A/B flow and COVID-19. **A** This section presents the number of positive samples for various pathogens as reported by both tNGS and TaqMan Array. The vertical axis indicates the number of samples, while the horizontal axis specifies the names of the etiological agents. **B** The pie chart on the left offers a visual comparison of the types of pathogens detected in each influenza-like patient sample between tNGS and TaqMan Array. The pie chart on the right illustrates pathogen types that are more frequently identified by tNGS compared to TaqMan Array among the 37 samples with a greater diversity of detected pathogens via tNGS. **C** This section details the total count of bacteria and fungi in patient samples with elevated C-reactive protein levels (exceeding the upper limit of normal) versus those with normal C-reactive protein levels. The comprehensive statistical results are provided in Supplemental Table 12

In this study, we compared targeted next-generation sequencing (tNGS) with the TaqMan Array Respiratory Tract Microbiota Comprehensive Card (Applied Biosystems™, A41238). Our tNGS detected two types of fungi, three types of viruses, and nine types of bacteria that were not included in the TaqMan Array’s target design. Notably, despite the TaqMan Array’s inclusion of rhinovirus, it failed to detect it, whereas tNGS did, with positive samples verified by Sanger sequencing, confirming the accuracy of tNGS results (Fig. 4A).

For the 13 viruses and 4 bacteria targeted by both methods, both TaqMan Array and tNGS detected six viruses at the same rate. However, tNGS outperformed TaqMan Array in detecting six viruses, and both methods showed high detection rates for bacteria (TaqMan: 72%, tNGS: 98%). To validate these findings, we used Sanger sequencing for samples with tNGS actual reads greater than 1000 or TaqMan Array reads less than 34. Out of 49 samples, tNGS detected pathogens in 12 samples that TaqMan Array missed, and identified more pathogens in 25 samples. Nine samples had identical results between both methods, while three samples showed discrepancies.

Further analysis revealed that among the 37 samples where tNGS detected more pathogens than TaqMan Array, 25 samples had more bacterial species detected by tNGS. This indicates that tNGS provides a more comprehensive bacterial detection than TaqMan Array (Fig. 4B). Additionally, the study found a correlation between pathogen detection and C-reactive protein levels, with higher pathogen counts observed in individuals with elevated C-reactive protein levels (Fig. 4C).

### Comparison of tNGS and mNGS

To evaluate tNGS, we compared it with mNGS results from alveolar lavage fluid samples. mNGS detected six species of fungi, three of which were outside tNGS’s target range. For the three fungi within tNGS’s target, tNGS outperformed mNGS in detection (*Candida albicans*: tNGS 6/49 vs. mNGS 5/49; *Aspergillus fumigatus*: tNGS 2/49 vs. mNGS 1/49; *Pneumocystis jirovecii*: tNGS 3/49 vs. mNGS 1/49). In viral identification, both methods yielded similar results for two viruses, but tNGS detected three additional cases of SARS-CoV-2.

A total of 15 bacterial species were identified, with tNGS successfully detecting 13, while two were excluded from our study design. Of the 13 bacteria encompassed by tNGS, 12 were consistently detected by both methods, and tNGS identified one additional case of *Pseudomonas aeruginosa* that mNGS missed (Extended Fig. 4A). Out of 49 samples analyzed, 36 had consistent pathogen profiles, and tNGS detected additional pathogens in seven samples (Extended Fig. 4B).

These results indicate a high degree of concordance between tNGS and mNGS, with tNGS either matching or outperforming mNGS in 87.8% of the samples. Notably, the utility of tNGS for pathogen detection significantly exceeded that of mNGS (Extended Fig. 4C). Consequently, tNGS demonstrated superior efficiency, requiring significantly less sequencing data compared to mNGS.

## Discussion

Various fields require targeted tNGS, such as specialized monitoring systems. The Global Influenza Surveillance Network is indispensable in the event of novel pathogen emergence, particularly during the early stages of the COVID-19 pandemic, where these networks played a crucial role in identifying atypical cases of influenza-negative Influenza-Like Illness (ILI) [22]. A notable attempt by the U.S. Centers for Disease Control and Prevention (CDC) is the Influenza SARS-COV-2 Multiplex Assay, which allows for comprehensive diagnosis of SARS-COV-2, influenza A, and/or influenza B infections through a single test [23]. However, it is crucial to note that many other microorganisms may exhibit similar clinical symptoms [24]. Therefore, our primary objective is to design surveillance tools capable of covering a broad spectrum of pathogens to maximize the efficiency of existing surveillance networks. The kits designed for influenza-like patients via UMPlex™ not only detect co-infections of multiple viruses but also evaluate other microorganisms in influenza-like illness and control groups. Extended Fig. 3B reveals that the infection rate of Human Herpesvirus 7 (HHV-7) is high across all groups, consistent with previous findings from studies isolating HHV-7 in saliva [25, 26]. In contrast, the infection rate of fungi is relatively low in both groups, while bacterial infections show significant variability. Previous studies have indicated that respiratory viral infections, such as influenza, predispose patients to bacterial co-infections, potentially exacerbating disease severity and increasing mortality [27]. For instance, early research frequently identified *Acinetobacter baumannii* as a common bacterial co-infection in critically ill patients [28]. However, our study, focusing primarily on outpatient populations, found a low co-infection rate of influenza A, particularly the H3N2 subtype, with *A. baumannii*, especially when compared to asymptomatic individuals and those infected with SARS-COV-2. These findings offer valuable insights for future research. The issue of co-infections between influenza viruses and bacteria is particularly significant, as existing methods cannot adequately assess the role of bacteria in viral infections. Bacterial co-infections are common in viral pneumonia: numerous reports suggest that approximately one-third of hospitalized patients with viral pneumonia have bacterial co-infections [29]. Some traditionally considered commensal bacteria have recently been identified as causative agents of pneumonia or bacterial/viral co-infections [30], posing significant challenges in pathogen diagnosis. Consequently, empirical antibiotic therapy is recommended for the majority of hospitalized patients with CAP [31]. The tNGS developed through the UMPlex™ design in this study provides indications of potential bacterial infections, which holds significant importance in antibiotic decision-making.

The principal impediment to the widespread implementation of tNGS resides in the intrinsic variability of amplification efficiency, a consequence of employing multiple primers. Within the realm of infectious disease diagnostics, the extensive heterogeneity of pathogens poses a formidable obstacle for tNGS in pathogen identification, owing to the substantial variability observed among pathogens, which underscores the potential for mutations within conserved gene regions. For instance, the World Health Organization (WHO) and the Centers for Disease Control and Prevention (CDC) have designated the matrix (M) gene as the optimal target for influenza A virus RT-PCR, owing to its high degree of sequence conservation[ [32] Nonetheless, the recent emergence of mutations within the M gene has engendered concerns regarding the sensitivity of influenza virus RT-PCR assays, thereby compromising their reliability for both influenza A(H1N1) and A(H3N2) subtypes [33]. Transcending traditional paradigms such as quantitative PCR (qPCR) and other limited-scope detection methodologies, tNGS emerges as a revolutionary approach capable of accommodating a multitude of targets within a single assay. In this investigation, we have adeptly addressed the challenge of missed detections attributable to variations in primer amplification efficiency. This was accomplished through the judicious utilization of multiple primer pairs targeting diverse genomic regions of the same pathogen. This innovative strategy endows our diagnostic tool with the requisite adaptability to navigate the perpetually evolving genomic landscape of pathogens, thereby offering a potent means to augment the precision and scope of pathogen detection. Notably, our method effectively mitigates the issue of clinical polymorphisms, ensuring robust and reliable identification of pathogens despite genetic variations.

A rigorous evaluation of our tNGS methodology, fortified with UMPlex™-designed primers, was conducted using a diverse array of clinical samples and virus cultures. Despite the modest scale of the collection, it was notably representative, encompassing influenza B Victoria and Yamagata lineages, which significantly contribute to global influenza-associated mortalities [34]. In terms of sensitivity, the samples derived from both culture isolates and clinical sources consistently demonstrated equivalent or superior performance compared to quantitative PCR (qPCR), as evidenced in Tables 3 and 4. This exceptional proficiency extends beyond influenza viruses to include a comprehensive array of other pathogens, such as viruses, bacteria, and fungi, as exemplified in Table 4 and Supplemental Table 11. For instance, sample 1046, initially identified as an influenza A infection via qPCR, was further revealed by the tNGS assay to harbor the Human Coronavirus 229E virus. Similarly, samples F94 and F95, previously confirmed for Severe Acute Respiratory Syndrome Coronavirus 2 (SARS-CoV-2) through qPCR, also exhibited the presence of Candida albicans, as disclosed by tNGS. While TaqMan array methods produced negative results, our tNGS analysis yielded positive findings. Specifically, samples 493, 484, 594, and 713 tested positive for Rhinovirus, sample 584 for human betaherpesvirus 5, and sample 528 for Human orthorubulavirus 2, with all results further validated through Sanger sequencing. These examples underscore the substantial diagnostic enhancement provided by tNGS assays compared to conventional qPCR and TaqMan array methods. The increased diagnostic yield, primarily attributed to the identification of potential pathogens that elude traditional techniques, represents a pivotal advantage.

To further elucidate the remarkable efficacy of our targeted Next-Generation Sequencing (tNGS) methodology, we undertook a comparative analysis with the classical metagenomic NGS (mNGS) approach in the context of pathogenic microorganism detection among adult pneumonia cases. Our empirical data unequivocally demonstrate that tNGS can seamlessly supplant mNGS in the majority of samples, particularly excelling in the identification of viral pathogens. This corroborates the conclusions derived from earlier commercial tNGS platforms. Although mNGS, in theory, possesses the potential to identify virtually all extant pathogens, its efficacy is substantially hampered by pervasive background noise, thereby undermining its practical clinical utility [35, 36]. In instances where both tNGS and mNGS converge on a common target, the pathogen detection achieved through tNGS aligns impeccably with our anticipated outcomes. As documented, the exclusion of the human gene background within mNGS could detrimentally impact the detection rates of Gram-negative bacteria, viruses, and intracellular bacteria, thereby exacerbating the risk of spurious negative results [37]. These empirical observations underscore the significant advantages of our proprietary tNGS procedure, which exhibits comparable performance metrics to those of leading commercial counterparts, thereby validating the versatility of our approach for broad applicability among researchers. Subsequent to pathogen identification via mNGS, the indispensable step of manual pathogen screening arises, mirroring the original disease manifestation as interpreted by mNGS. This highlights the imperative of developing products that harmonize with individual research objectives. By tailoring pathogen selection to align with discrete application scenarios, the necessity for manual interpretation can be effectively eradicated. Additionally, tNGS demands a significantly reduced data volume, suggesting the potential for substantial cost savings in sequencing endeavors.

In our evaluation of UMPlex™, we have pinpointed limitations. We aim to establish a holistic platform that is freely accessible to all users, and have developed the website at http://183.11.230.224:8043/. However, we found that the necessary resources are considerable, thereby constraining the capacity to support only a limited number of clients. Nevertheless, we have made the primer design process and validation methodologies publicly available. Any individual can now execute this procedure on a local server.

In summary, this research demonstrates the viability of our tailored tNGS solution and highlights our adept handling of challenges posed by template mutations through the strategic use of multiple primer pairs. This showcases our innovative and practical approach. The implementation of the tNGS assay has the potential to significantly enhance diagnostic accuracy and surveillance, leveraging contemporary influenza point-of-care testing methodologies for more profound etiological investigations. We remain optimistic that this approach will empower individuals to develop personalized tNGS systems, thereby equipping healthcare professionals with essential tools for adaptable and effective strategies.

This article utilized Wetab AI Pro, version AI-4.0, to refine grammar and rectify spelling errors.

## Supplementary Information

Supplementary Material 1: Extended Fig. 1. Amplification Efficiency of Culture Samples. A and B: The relationship between CT values and the number of reads associated with primers targeting Influenza A virus. C and D: The relationship between CT values and the number of reads associated with primers targeting *S. aureus*. To enhance clarity and facilitate comparison, the CT values have been rounded; the precise CT values are available in Supplemental Table 7 (Sheet 2). Extended Fig. 2. Comparison of the Highest Pathogenic Reads. This figure delineates a comparison of the highest counts of pathogenic reads identified by tNGS across two distinct cohorts: the ‘Influenza-Like Patient’ group and the ‘Asymptomatic Control’ group. The former includes patients with confirmed pathogens, while the latter functions as a control, offering essential insights into pathogen detection within these populations. Extended Fig. 3. Comparison of Pathogen Detection Proportions. This figure presents a comparative analysis of pathogen detection proportions in samples with confirmed pathogens (not including known pathogens) versus those identified in the healthy control group. A: The detection rates of fungi across different groups. B: Illustrates the detection rates of the three most prevalent viruses (excluding those that clearly cause influenza-like illnesses) among the groups. C: The detection rates of the three most common bacteria across the groups. D: The prevalence of *Acinetobacter baumannii* within the various cohorts. The corresponding actual reads can be found in Supplemental Table 11. Extended Fig. 4. Comparison of tNGS and mNGS. This figure contrasts the efficacy of tNGS and mNGS in the detection of pathogens. A: The number of positive samples for different pathogens as reported by both tNGS and mNGS. The vertical axis represents the sample count, while the horizontal axis denotes the respective etiologies. B: The diversity of pathogen types identified in each patient sample by tNGS and mNGS. C: The proportion of pathogen reads relative to the total data for both tNGS and mNGS. The actual read counts are available in Supplemental Table 13.

Supplementary Material 2.

## Data Availability

No datasets were generated or analysed during the current study.
