# Supplementary figures and images for: UMPlex™: a targeted next-generation sequencing primer design workflow
Source: Virol J. 2025 Jul 5;22:222. doi: 10.1186/s12985-025-02831-6 (PMC12228999; doi:10.1186/s12985-025-02831-6)

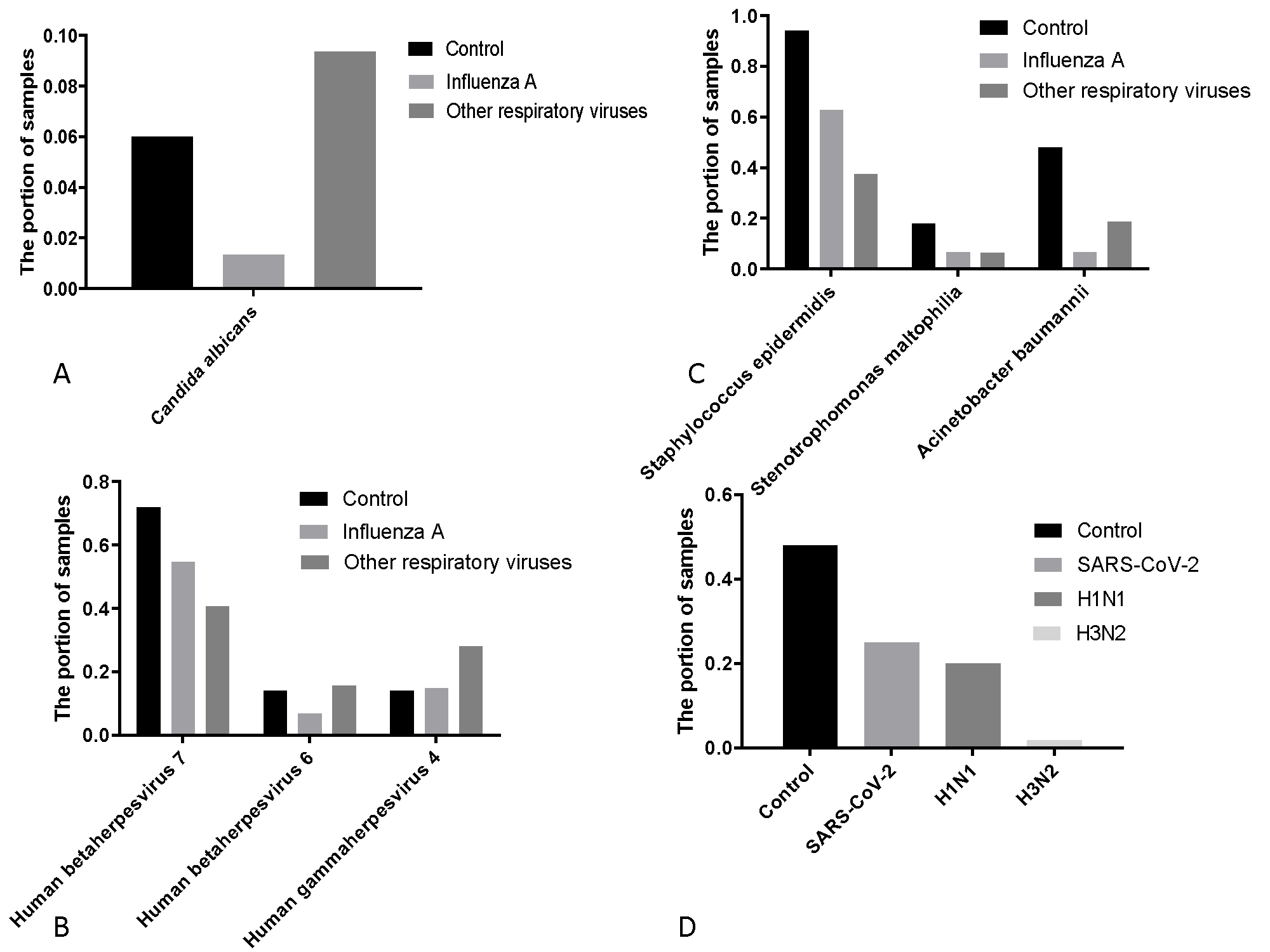

Supplement: Supplementary file 1 — Supplementary Material 1: Extended Fig. 1. Amplification Efficiency of Culture Samples. A and B: The relationship between CT values and the number of reads associated with primers targeting Influenza A virus. C and D: The relationship between CT values and the number of reads associated with primers targeting S. aureus. To enhance clarity and facilitate comparison, the CT values have been rounded; the precise CT values are available in Supplemental Table 7 (Sheet 2). Extended Fig. 2. Comparison of the Highest Pathogenic Reads. This figure delineates a comparison of the highest counts of pathogenic reads identified by tNGS across two distinct cohorts: the ‘Influenza-Like Patient’ group and the ‘Asymptomatic Control’ group. The former includes patients with confirmed pathogens, while the latter functions as a control, offering essential insights into pathogen detection within these populations. Extended Fig. 3. Comparison of Pathogen Detection Proportions. This figure presents a comparative analysis of pathogen detection proportions in samples with confirmed pathogens (not including known pathogens) versus those identified in the healthy control group. A: The detection rates of fungi across different groups. B: Illustrates the detection rates of the three most prevalent viruses (excluding those that clearly cause influenza-like illnesses) among the groups. C: The detection rates of the three most common bacteria across the groups. D: The prevalence of Acinetobacter baumannii within the various cohorts. The corresponding actual reads can be found in Supplemental Table 11. Extended Fig. 4. Comparison of tNGS and mNGS. This figure contrasts the efficacy of tNGS and mNGS in the detection of pathogens. A: The number of positive samples for different pathogens as reported by both tNGS and mNGS. The vertical axis represents the sample count, while the horizontal axis denotes the respective etiologies. B: The diversity of pathogen types identified in each patient sample by tN [file 12985_2025_2831_MOESM1_ESM.zip › Supplemental Figure 3 .tif]

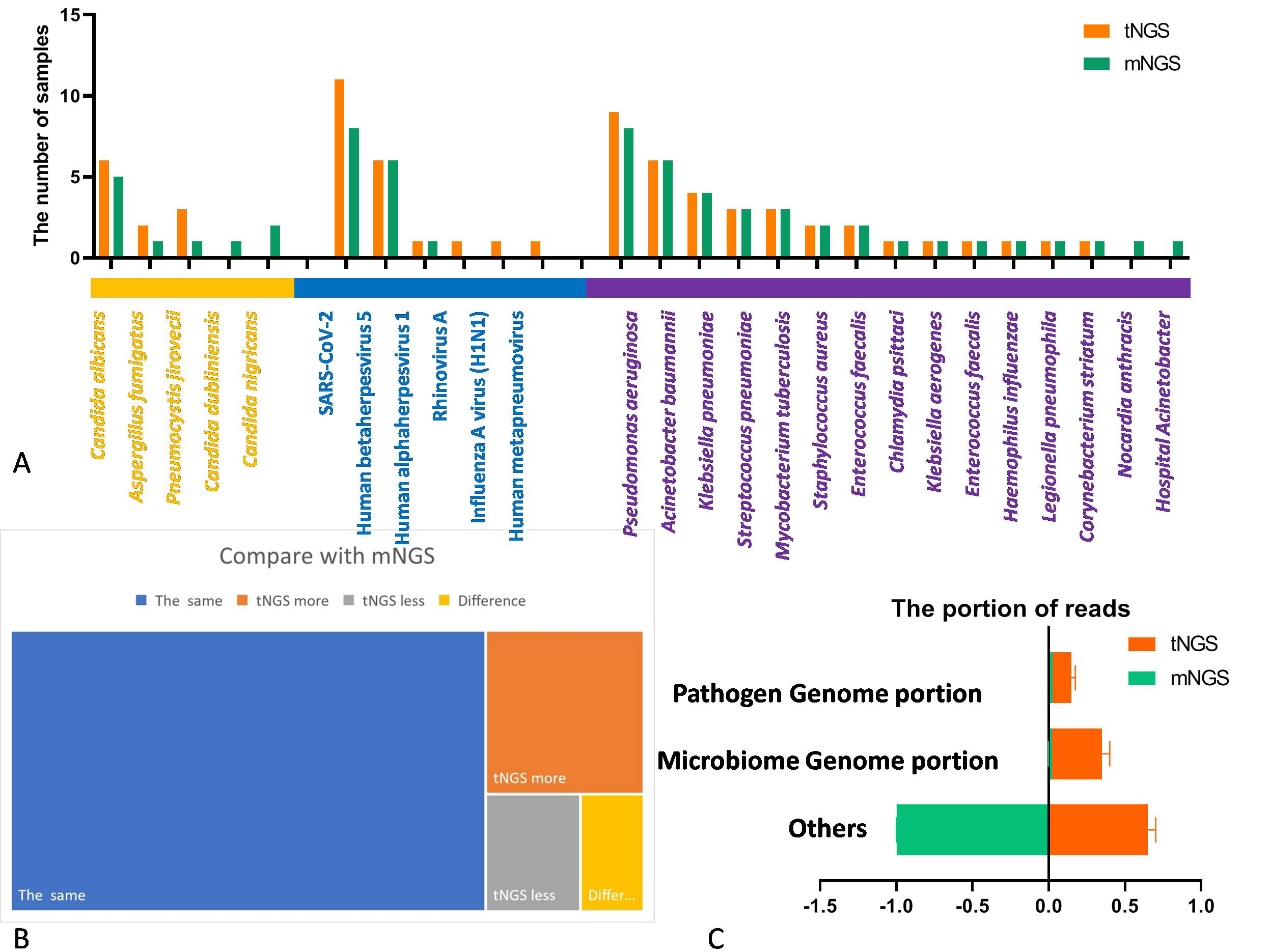

Supplement: Supplementary file 1 — Supplementary Material 1: Extended Fig. 1. Amplification Efficiency of Culture Samples. A and B: The relationship between CT values and the number of reads associated with primers targeting Influenza A virus. C and D: The relationship between CT values and the number of reads associated with primers targeting S. aureus. To enhance clarity and facilitate comparison, the CT values have been rounded; the precise CT values are available in Supplemental Table 7 (Sheet 2). Extended Fig. 2. Comparison of the Highest Pathogenic Reads. This figure delineates a comparison of the highest counts of pathogenic reads identified by tNGS across two distinct cohorts: the ‘Influenza-Like Patient’ group and the ‘Asymptomatic Control’ group. The former includes patients with confirmed pathogens, while the latter functions as a control, offering essential insights into pathogen detection within these populations. Extended Fig. 3. Comparison of Pathogen Detection Proportions. This figure presents a comparative analysis of pathogen detection proportions in samples with confirmed pathogens (not including known pathogens) versus those identified in the healthy control group. A: The detection rates of fungi across different groups. B: Illustrates the detection rates of the three most prevalent viruses (excluding those that clearly cause influenza-like illnesses) among the groups. C: The detection rates of the three most common bacteria across the groups. D: The prevalence of Acinetobacter baumannii within the various cohorts. The corresponding actual reads can be found in Supplemental Table 11. Extended Fig. 4. Comparison of tNGS and mNGS. This figure contrasts the efficacy of tNGS and mNGS in the detection of pathogens. A: The number of positive samples for different pathogens as reported by both tNGS and mNGS. The vertical axis represents the sample count, while the horizontal axis denotes the respective etiologies. B: The diversity of pathogen types identified in each patient sample by tN [file 12985_2025_2831_MOESM1_ESM.zip › Supplemental figure 4.tiff]
