# Supplementary material for: UMPlex™: a targeted next-generation sequencing primer design workflow
Source: Virol J. 2025 Jul 5;22:222. doi: 10.1186/s12985-025-02831-6 (PMC12228999; doi:10.1186/s12985-025-02831-6)
Supplement: Supplementary file 2 — Supplementary Material 2. [file 12985_2025_2831_MOESM2_ESM.zip › Supplemental Table 8 Reproducibility.docx]

**Supplemental Table 8: Amplification Reproducibility of Culture Samples**

| Assay | | Within batch reproducibility(n=5) | | | Reproducibility among batches(n=5) | | |
| --- | --- | --- | --- | --- | --- | --- | --- |
|  |  | Mean | Std. Deviation | %CV* | Mean | Std. Deviation | %CV* |
| Influenza A | Pc-13-High | 1477 | 375.2 | 25.39274 | 1552 | 409.4 | 26.37356 |
|  | Pc-13-Low | 179.4 | 26.88 | 14.98085 | 192.4 | 26.23 | 13.63095 |
|  | Pc-2-High | 535.2 | 61.78 | 11.54249 | 517.4 | 71.18 | 13.75685 |
|  | Pc-2-Low | 56.8 | 23.38 | 41.16482 | 55.8 | 26.42 | 47.3539 |
| *S.aureus* | Pc-16-High | 51459 | 6799 | 13.21301 | 54375 | 7418 | 13.64145 |
|  | Pc-16-Low | 2425 | 430.4 | 17.74726 | 2355 | 477.2 | 20.26456 |
|  | Pc-17-High | 22593 | 7616 | 33.70993 | 23562 | 8009 | 33.99258 |
|  | Pc-17-Low | 1506 | 251.6 | 16.70164 | 1442 | 306.3 | 21.24247 |

*: %CV: Coefficient of variation

The corresponding actual reads are available in Supplementary Table 9.
